
JOURNAL INFORMATION
==============================
NLM Title Abbreviation: Inter Econ
Journal ID: Inter Econ
NLM Journal ID: 101086399

Inter Economics

ISSN: 0020-5346

ARTICLE INFORMATION
==============================
PMCID: PMC8664671
PMID: 34924593
DOI: 10.1007/s10272-021-1004-7
Article ID: 1004
Article version: 1
Subjects: Editorial

The Glasgow Climate Pact: A Robust Basis for the International Climate Regime in the 2020s

Michaelowa Axel axel.michaelowa@pw.uzh.ch

grid.7400.3 0000 0004 1937 0650 Institute of Political Science, University of Zurich, Affolternstrasse 56, 8050 Zürich, Switzerland
Electronic publication date: 2021 Dec 11
Print publication date: 2021
Volume: 56
Issue: 6
First page: 302
Last page: 303
Copyright: © The Author(s) 2021
License: Open Access: This article is distributed under the terms of the Creative Commons Attribution 4.0 International License (https://creativecommons.org/licenses/by/4.0/).
Open Access funding provided by ZBW — Leibniz Information Centre for Economics.
License URL: https://creativecommons.org/licenses/by/4.0/

issue-copyright-statement: © The Author(s) 2021

==============================

With close to 40,000 participants, COP26 in Glasgow was the largest UN climate conference ever, followed by Paris in 2015 and Copenhagen in 2009. This showed the appetite of the global climate policy community to engage in a large in-person event after nearly two years of the COVID-19 pandemic. While a number of logistical issues emerged during the first week of the conference, the process ran smoothly in the second week despite a resurgence of COVID-19 cases towards the end. The large number of participants led to a venue closure one day when the capacity limit of 10,000 was reached, and to restricted access to the negotiations, where each party could only send one negotiator per room, and each observer constituency could send only one observer. The otherwise impeccable UK diplomacy was thereby able to thwart reproaches for not being inclusive.

The COP26 agenda had a number of key themes: international climate finance, mitigation ambition, the finalisation of the rulebook on international market mechanisms (Article 6 of the Paris Agreement), detailed approaches to the reporting of national emissions and progress in reaching national emissions pledges (nationally determined contributions, NDCs), and common time frames for NDCs. Adaptation, loss and damage caused by climate change impacts were also sharply in focus.

In the run-up to COP26, the UK COP26 presidency worked to secure side deals on the four topics “coal, cars, cash and trees”; this was added to the previously stated aim of reducing methane by 30% between 2020 and 2030.

There was great public pressure on COP26 to produce a strong outcome, with over 100,000 people demonstrating in Glasgow on 6 November, reinforced by the Sixth Assessment Report of the Intergovernmental Panel on Climate Change published in August stating that warming has already reached 1.1 degrees Celsius. The graph showing a sea level rise of several metres by 2300 – even under low emissions scenarios – woke up many people. On the other hand, the continued pandemic, the increased rivalry between the US and China and other geopolitical tensions were forces working against a strong result at the Glasgow conference.

Emulating the successful approach in Paris, the UK Presidency scheduled the heads of state segment for the first few days of COP. This led to a positive dynamic regarding ambition and finance, as numerous mitigation and finance pledges were made. The Adaptation Fund received US $356 million, and the Least Developed Country Fund US $413 million. The US $8.5 billion Just Energy Transition programme of developed countries for coal transition in South Africa could become a blueprint for similar programmes in other coal-rich countries.

The unexpected announcement of net zero targets by key countries of the Arabian Peninsula in the week before COP26 created a welcome dynamic, with the United Arab Emirates announcing a 2050 target date, Saudi Arabia and Bahrain settling for 2060. At COP itself, the developing country giants India and Nigeria followed with 2070 and 2060 target dates, respectively. This means that 89% of global emissions are covered by net zero targets. The International Energy Agency has estimated that global warming can be limited to 1.8 degrees Celsius due to the new pledges, while the NGO-backed Climate Action Tracker still sees a temperature increase of 2.4 degrees Celsius.

A surprise that changed the dynamics and was crucial for a positive COP26 outcome was the US-China declaration on 10 November, which clearly stated that the two countries wanted a successful outcome on the Paris Rulebook, that China would strengthen its NDC in the next year and that Chinese coal use would peak in 2025, not 2030. A similar declaration in 2014 had paved the way for China to give up on a sharp divide between industrialised and developing countries and thus enabled the Paris Agreement of 2015.

At COP26, the United Nations Framework Convention on Climate Change Secretariat and the UK Presidency cooperated well, leading to the smooth publication of negotiation texts in rapid succession. A novel feature was the avoidance of "all-night" sessions by the organisers who announced at 8 p.m. on 12 November that the final text would be available at 8 a.m. the following morning. This certainly contributed to a better outcome in the final plenary.

Brazil and Russia – as well as smaller countries like Bolivia, Nicaragua and Venezuela that had often put spokes in the wheels of COPs – had a very constructive attitude this time. A moment of high drama in the final plenary saw various "huddles" involving the African Group who wanted to ensure adaptation funding through Article 6.2 and China and India's revision of the wording on coal subsidy reduction or elimination. US climate envoy John Kerry masterfully worked the room and ensured that all negotiation threads were followed in a coordinated manner. While India and China reopened the text in order to talk about a phase-down of coal subsidies instead of a phase-out, this did not unravel the finely crafted balance and the package of decisions could be adopted by the evening of 13 November, "just" one day after the scheduled close of the conference.

The cover decisions, which included the contentious coal subsidy language, generated significant media attention but are not relevant for the actual implementation of the Paris Agreement. The key language from these decisions is the call for a further update of the NDCs in 2022.

International carbon markets through "cooperative approaches" and the Article 6.4 mechanism can now be implemented, with developing countries benefitting from capacity building support. Double bookkeeping ("corresponding adjustments") is to be done for all transactions. Non-greenhouse gas metrics are generally allowed. Developed countries do not have to pay an adaptation tax on Article 6.2 transactions. For Article 6.4, corresponding adjustments depend on the authorisation of transactions by the host country. An adaptation tax of 5% will be paid, and 2% of credits will be cancelled to achieve an overall mitigation in global emissions. All ongoing projects from the Clean Development Mechanism (CDM) can be transitioned, as well as emissions credits from such projects registered from 2013 onwards. Baseline methodologies for the calculation of emissions credits are stringent, and testing of the additionality of activities is required to prevent business-as-usual activities from receiving credits. Thus, stringent rules were combined with a lenient CDM transition.

Regarding transparency, reporting tables are mandatory for all parties but flexibility exists in filling specific cells. Confidentiality can be invoked. The review can trigger action by the compliance committee in the case of significant and persistent inconsistencies.

Common timeframes are defined by the end dates of NDCs – they will be aligned in five year periods, e.g. 2035, 2040, 2045. Regarding climate finance, developed countries were asked to double adaptation funding between 2019 and 2025, but no binding target has been set. A dialogue on funding of loss and damage will be held by 2024. The negotiations on the post-2025 collective quantified goal on climate finance will be done through an ad hoc working programme with four meetings per year, with the deadline in 2024. A two-year Glasgow–Sharm el-Sheikh work programme will be held on the Global Goal for Adaptation and address adaptation metrics. Overall, COP26 has established a firm basis for the implementation of the Paris Agreement. The short-term emissions gap still remains but these steps will help to make it smaller.

Axel Michaelowa, University of Zurich, Switzerland; and Perspectives Climate Group, Freiburg, Germany.

